# 

**Published:** 1903-03-14

**Authors:** 


### PUBLISHER'S NOTICE.

TO SUBSCRIBERS AND READERS OF "THE HOSPITAL."

### THE PRICE OF "THE HOSPITAL."

The circulation of The Hospital is now so large as to make it essential that the price shall approximate more closely to the actual cost of production than it has done in the past. It is therefore found necessary, after full consideration, to increase the price of this Journal **from 2d. to 3d. per copy**. This change in price will take effect from the first number of Volume XXXIV., which will be issued on Saturday, April 4th, 1903. The terms of subscription from that date will be as follows:—

| | Great Britain. (Post free.) | Foreign and Colonial. (Post free.) |
|---|---|---|
| For one year ... ... | 15s. 0d. | 19s. 6d. |
| For six months ... ... | 7s. 6d. | 10s. 0d. |
| For three months ... | 3s. 9d. | 5s. 0d. |

| Monthly Parts. | Post free to any part of the World. |
|---|---|
| For one year ... ... ... | 15s. 6d. |
| For six months ... ... ... | 7s. 9d. |
| For three months ... ... ... | 4s. 0d. |

N.B.—Subscribers who have already paid their subscriptions will continue to receive The Hospital as issued at the old rates until such subscriptions expire.

**All new Subscriptions commencing on and after March 7, 1903, will be charged at the above Rates.**

Publishing and Editorial Offices, "The Hospital" Buildings, 28 & 29 Southampton Street, Strand, London, W.C.

### THE STUDENT OF MEDICINE.

### APPOINTMENTS.

**J. M. Barbour**, M.B., appointed Medical Officer of Health for Ramsey, Isle of Man. **G. A. H. Barton**, M.D.Brux., L.R.C.P.-Lond., M.R.C.S., L.S.A., appointed Anæsthetist to the Female Lock Hospital. **J. P. Drinkwater**, M.R.C.S.Eng., reappointed Medical Officer of Health to the Llangollen Urban District Council. **Hugh F. Hine**, M.B.Lond., F.R.C.S.Eng., appointed Honorary Surgeon to the Newark-on-Trent Hospital. **W. Nolan**, L.R.C.S.I., L.R.C.P.&L.M., appointed Clinical Assistant to the Chelsea Hospital for Women. **H. Reginald Pring**, M.R.C.S., L.R.C.P.,

L.D.S.Eng., appointed Honorary Dental Surgeon to the City of London Asylum. **Ernest Ringrose**, M.R.C.S., L.R.C.P.Lond., appointed Honorary Surgeon to the Newark on-Trent Town and District Hospital. **Harry Stallard**, B.A.Cantab., M.R.C.S., L.R.C.P.Lond., appointed Honorary Surgeon to the Newark-on-Trent Town and District Hospital. **E. Taunton**, M.R.C.S., L.R.C.P.Lond., appointed First Assistant Medical Officer to the Whitechapel Union Infirmary. **Arthur John Wallace**, M.D.-Edin., C.M., appointed Surgeon to the Hospital for Women, Liverpool. **Spencer Wicks**, B.A., M.R.C.S., L.R.C.P., appointed Medical Officer to the Premier, Bultfontein, and Dutoitspan Mines, Kimberley, S. Africa. **James Hayward Willett**, M.B., Ch.B.Vict., appointed Assistant Surgeon to the Hospital for Women, Liverpool. **Thomas Wilson**, M.D., F.R.C.S., appointed Honorary Obstetric Officer to the General Hospital, Birmingham.

St. Thomas's Hospital.—The following gentlemen have been selected as House Officers, from Tuesday, March 3rd, 1903:—House Physicians: **C. N. Sears**, L.R.C.P., M.R.C.S.; **A. E. Boycott**, M.A., M.B., B.Ch.Oxon., B.Sc.Oxon.; Assistant House Physicians: **O. Hildesheim**, B.A., M.B., B.Ch.Oxon., L.R.C.P., M.R.C.S.; **H. W. Sexton**, L.R.C.P., M.R.C.S. House Surgeons: **J. W. Rob**, B.A., M.B., B.C.Cantab.; **T. B. Henderson**, M.A., M.B., B.Ch.Oxon.; **J. P. Hedley**, M.A., M.B., B.C.Cantab.; **A. B. Bradford**, M.B., B.S.Durh., L.R.C.P., M.R.C.S.; Assistant House Surgeons: **J. E. Adams**, L.R.C.P., M.R.C.S.; **H. Upcott**, L.R.C.P., M.R.C.S.; **C. Wheen**, B.A.Oxon., L.R.C.P., M.R.C.S.; **N. Carpmael**, L.R.C.P., M.R.C.S. Obstetric House Physicians: (*Senior*) **G. A. C. Shipman**, M.A., M.B., B.C.Cantab., L.R.C.P., M.R.C.S.; (*Junior*) **W. M. C. Glanville**, B.A., M.B., B.Ch.-Oxon. Ophthalmic House Surgeons: (*Senior*) **A. E. A. Loosely**, B.A.Oxon., L.R.C.P., M.R.C.S.; (*Junior*) **A. C. Hudson**, M.A., M.B., B.C.Cantab. Clinical Assistants in the Special Department for Diseases of the Throat: **R. E. H. Leach**, B.A.Oxon., L.R.C.P., M.R.C.S. Clinical Assistants in the Special Department for Diseases of the Skin: **T. Guthrie**, B.A.Cantab., L.R.C.P., M.R.C.S; **W. M. Strong**, M.A., B.C.Cantab., L.R.C.P., M.R.C.S. Clinical Assistant in the Special Department for Diseases of the Ear: **A. Bevan**, M.B.Lond., L.R.C.P., M.R.C.S. Clinical Assistant in the Electrical Department and in X-Ray Department: **W. M. Strong**, M.A., B.C.Cantab., L.R.C.P., M.R.C.S. Several other gentlemen have received extensions of their appointments.

**"The Hospital" Institutional Library.**

"Hospitals and Asylums of the World." 4 Vols., with a Portfolio of Plans, complete £12 12s.

"Burdett's Hospitals and Charities, 1902." 5s.

"Cottage Hospitals, General, Fever, and Convalescent." 10s. 6d.

"Hospital Expenditure: The Commissariat." 2s. 6d.

"A Legal Handbook for the Use of Hospital Authorities." 2s. 6d.

"The Cottage Hospital Case Book or Register of Patients." 10s. and 12s. 6d.

"The Furnishing and Appliances of a Cottage Hospital." 6d.

All these are published by the Scientific Press, Ltd., and may be obtained through any bookseller, or direct from the publishers, 28 and 29 Southampton Street, Strand, London, W.C.

### PUBLISHER'S NOTICE.

TO SUBSCRIBERS AND READERS OF "THE HOSPITAL."

### THE PRICE OF "THE HOSPITAL."

The circulation of The Hospital is now so large as to make it essential that the price shall approximate more closely to the actual cost of production than it has done in the past. It is therefore found necessary, after full consideration, to increase the price of this Journal **from 2d. to 3d. per copy**. This change in price will take effect from the first number of Volume XXXIV., which will be issued on Saturday, April 4th, 1903. The terms of subscription from that date will be as follows:—

| | Great Britain. (Post free.) | Foreign and Colonial. (Post free.) |
|---|---|---|
| For one year ... ... | 15s. 0d. | 19s. 6d. |
| For six months ... ... | 7s. 6d. | 10s. 0d. |
| For three months ... | 3s. 9d. | 5s. 0d. |

| Monthly Parts. | Post free to any part of the World. |
|---|---|
| For one year ... ... ... | 15s. 6d. |
| For six months ... ... ... | 7s. 9d. |
| For three months ... ... ... | 4s. 0d. |

N.B.—Subscribers who have already paid their subscriptions will continue to receive The Hospital as issued at the old rates until such subscriptions expire.

**All new Subscriptions commencing on and after March 7, 1903, will be charged at the above Rates.**

Publishing and Editorial Offices, "The Hospital" Buildings, 28 & 29 Southampton Street, Strand, London, W.C.

### STANDARD NURSING MANUALS.

### A HANDBOOK FOR NURSES.

By J. K. Watson, M.D., M.B., C.M.

Second Edition. Crown 8vo. over 400 pp., profusely illustrated, **5s.** net, **5s. 5d.** post free.

"A concise and comprehensive exposition of as much medical knowledge as a nurse needs. The work cannot but prove useful to those for whom it has been designed." *Scotsman.*

### ELEMENTARY ANATOMY AND SURGERY FOR NURSES.

By William McAdam Eccles, M.S.Lond., M.B., &c.

Crown 8vo. Illustrated, cloth, **2s. 6d.** post free.

"Valuable to nurses who are beginning the study of anatomy. The instruction is given very clearly and concisely, and the reader is able to glean much information in a very condensed form."—*Nursing Notes.*

### NURSING: ITS THEORY AND PRACTICE.

By Percy G. Lewis, M.D., M.R.C.S., L.S.A.

New and Enlarged Edition.

Crown 8vo. over 400 pp. profusely illustrated, **3s. 6d.** post free.

"Nurses will find the book full of interest and instruction, which cannot fail to assist them in their work." *British Medical Journal.*

### ELEMENTARY PHYSIOLOGY FOR NURSES.

By C. F. Marshall, M.D., B.Sc., F.R.C.S.

Crown 8vo. Illustrated, cloth, **2s.** post free.

"Nurses will find in it all that they require to know of the subjects treated."—*Daily Chronicle.*

### NURSING: ITS PRINCIPLES AND PRACTICE.

By Isabel Adams Hampton.

New and Enlarged Edition.

Second Edition. Crown 8vo. illustrated, **7s. 6d.** net, post free **7s. 10d.**

"The subject has been scientifically, carefully, and completely treated by Miss Hampton."—*The Hospital.*

### MIDWIVES' POCKET BOOK.

(With chapter on the Midwives Bill.)

By Honnor Morten.

Small crown 8vo. cloth, **1s.** post free.

An exceedingly useful handbook for those entering for the L.O.S. certificate as well as for qualified Midwives.

### OPHTHALMIC NURSING.

By Sydney Stephenson, M.B., F.R.C.S.E.

Second Edition. Crown 8vo. illustrated, with glossary of terms, cloth, **3s. 6d.** net, **3s. 10d.** post free.

"May very advantageously be studied by nurses who are about to enter the Ophthalmic service of a hospital." *Lancet.*

### ART OF MASSAGE.

By A. Creighton Hale.

Second Edition. Demy 8vo. Profusely illustrated, cloth gilt, **6s.** post free.

"Mrs. Hale seems to have had considerable experience, not only as a practitioner, but as a teacher of the art, and explains in a series of clearly illustrated articles the many complaints for which massage has been found beneficial, and the special manipulations required in each case." *Morning Post.*

### NURSING IN DISEASES OF THE THROAT, NOSE AND EAR.

By P. Macleod Yearsley, F.R.C.S.Eng., M.R.C.S., L.R.C.P.Lond.

Demy 8vo. Illustrated, cloth, **2s. 6d.** post free.

"Of practical service to every nurse who wishes to be thoroughly trained." *Scottish Medical and Surgical Journal.*

### ART OF FEEDING THE INVALID.

By a Medical Practitioner and a Lady Professor of Cookery.

New and Popular Edition. Demy 8vo. cloth, **1s. 6d.** post free.

"A new edition of this valuable work is very welcome, and we heartily commend it to all who desire reliable and practical instruction in sick cookery."—*Nursing Notes.*

### SURGICAL WARD-WORK AND NURSING.

By Alexander Miles, M.D.Edin., C.M., F.R.C.S.E.

Revised Edition. Demy 8vo. Illustrated, cloth gilt, **3s. 6d.** net, **3s. 10d.** post free.

"An illustrated manual which will furnish much needed guidance to the student and the nurse in the early stages of their apprenticeship."—*The Times.*

### HOSPITAL SISTERS AND THEIR DUTIES.

By Eva C. E. Lückes, Matron to the London Hospital.

Third Edition. Crown 8vo. Cloth gilt, **2s. 6d.** post free.

"An admirable guide to the beginner anxious to take up a Sister's work. Its pages bring help and knowledge imparted in a very sympathetic, straightforward manner by one who has had practical experience in the lesson she teaches."—*Gentlewoman.*

*COMPLETE CATALOGUE POST FREE ON APPLICATION.*

The SCIENTIFIC PRESS, LTD., 28 & 29 Southampton Street, Strand, London, W.C.

